# Assessment of Pet Owners’ Knowledge on Parasitic Infection in Sylhet City Corporation, Bangladesh

**DOI:** 10.1155/japr/3031689

**Published:** 2026-04-15

**Authors:** Saiful Islam, Rakibul Hasan, Shofiqul Islam, Md. Khairul Amin Rafi, Md. Saruar Jahan Nayem, Onnapurna Roy, Tushar Kanti, Marjana Mowrin Jui, Muhammad Mujahidul Islam, Mohammad Sujaur Rahman, Md. Mowdudul Hasan Talha, Real Datta, Kazi Mehetazul Islam, Saiful Islam

**Affiliations:** ^1^ Department of Anatomy & Histology, Sylhet Agricultural University, Sylhet, Bangladesh, sau.ac.bd; ^2^ Department of Microbiology & Immunology, Sylhet Agricultural University, Sylhet, Bangladesh, sau.ac.bd; ^3^ Faculty of Veterinary, Animal and Biomedical Sciences, Sylhet Agricultural University, Sylhet, Bangladesh, sau.ac.bd; ^4^ Sylhet Pet Care, Sylhet, Bangladesh; ^5^ University of Arkansas at Little Rock, Little Rock, Arkansas, USA, ualr.edu; ^6^ Department of Pharmacology and Toxicology, Sylhet Agricultural University, Sylhet, Bangladesh, sau.ac.bd; ^7^ Department of Parasitology, Sylhet Agricultural University, Sylhet, Bangladesh, sau.ac.bd

**Keywords:** dogs, ESCCAP, intestinal parasites, parasite control, risk assessment, zoonosis

## Abstract

Pets can contain parasites along with other infectious diseases. This survey investigates risk factors associated with pet owners’ sociodemographic status and categorizes pet animals into different risk groups, as reported by their owners, in Sylhet City Corporation, Bangladesh. Data were collected using a preplanned questionnaire from cat and dog owners at different pet clinics. The responses provided details on pets’ living conditions and classified them into one of the four levels of risk for ESCCAP infections (A, B, C, and D). The chi‐square test examined associations between risk groups and the owners’ sociodemographic factors. This study assessed 197 cat owners and 32 dog owners to assess their pets’ risk of diseases using ESCCAP guidelines and its relationship with owners’ sociodemographic factors. Among dogs, 50% were classified in the highest‐risk group (D), requiring monthly deworming, while 54% of cats were in the lowest‐risk group (A), reflecting reduced exposure to parasites. For dogs, significant associations were observed between risk groups and owners’ education, gender, veterinary visits, and residency (*p* < 0.05). Among cat owners, owners’ residency, responsibility, vet visits, and attitude toward pets are significantly associated with different risk groups (*p* < 0.05). Deworming compliance was higher among cat owners (55.83%) than dog owners (18.75%), though it remained suboptimal overall. Awareness of zoonotic diseases was low, with only 21.87% of dog owners and 25.38% of cat owners informed. The serious shortage of zoonotic awareness among pet owners forms the basis of the One Health challenges. This represents a major threat to public health owing to the intimate relationship between owners and vulnerable pets, such as 50% of dogs in group D. Vaccination rates were higher for cats (56.34%) than dogs (28.12%). Pets in urban areas faced lower risks than those in rural settings (*p* < 0.001), underscoring the role of environmental exposure. These findings emphasize the urgent need for comprehensive health education, better veterinary engagement, and targeted interventions to enhance parasite control and reduce zoonotic risks within the One Health framework.

## 1. Introduction

Parasitic infections in companion animals, such as dogs and cats, are caused by ectoparasites (ticks, fleas, and mites) and endoparasites (roundworms and tapeworms), posing veterinary and zoonotic risks worldwide, including Bangladesh [1, 2]. Transmission occurs via contaminated feces, food, or skin penetration, with notable zoonotic parasites like *Toxoplasma gondii* causing serious health issues [3, 4]. Common parasites include *Toxocara* spp., *Ancylostoma* spp., and protozoa like *Giardia* and *Isospora*, often leading to gastrointestinal symptoms and compromised health [5, 6]. Parasitic infections in pets have significant implications for human and animal health, necessitating effective prevention and control measures [7]. The prevalence and severity of these infections are influenced by geography, climate, host factors (e.g., age, immunity, and breed), and management practices like deworming [8, 9]. Young animals are particularly vulnerable to infections such as *Toxocara* spp., while free‐roaming animals face higher risks than household pets [10]. Studies in Bangladesh highlight concerning rates of parasitic infections: ectoparasites in semiurban dogs and cats (56.92% and 76.92%, respectively) and gastrointestinal parasites like *Toxocara cati* and *Ancylostoma tubaeforme* [11, 12]. Stray dogs show significantly higher parasite prevalence than pets, with local breeds being more affected [13]. Pets offer companionship and emotional support but are associated with over 60 zoonotic agents, posing public health risks [14, 15]. Common zoonotic parasites include *Dirofilaria immitis*, *Thelazia callipaeda*, and *Angiostrongylus vasorum* [16]. Significant prevalence rates of gastrointestinal parasites in companion animals have been demonstrated in recent studies published in Bangladesh. For example, a study carried out in urban environments discovered that at least one gastrointestinal parasite, including zoonotic species like *Toxocara*, *Ancylostoma*, and *Trichuris* spp., infected 63.3% of dogs and 58.4% of cats [17]. Similarly, molecular research in Bangladesh found *Cryptosporidium* spp. in 25% of dogs and 29.9% of cats and *Giardia duodenalis* in 8.1% of cats and 4.2% of dogs, highlighting the zoonotic risk associated with domestic pets [18]. Companion animals often have ectoparasites in addition to endoparasites. According to a study carried out in Sylhet City, fleas or ticks, especially *Ctenocephalides* species, affected 35.1% of the dogs and cats that were tested, as well as *Rhipicephalus sanguineus*, both of which are zoonotic disease vectors [19]. The outcomes reveal that companion animals in Bangladesh could act as human‐infecting parasite reservoirs. Despite this possible risk, little is known about pet owners’ awareness and knowledge of parasite illnesses, particularly in places like Sylhet City Corporation that are quickly urbanizing. Effective control measures such as routine deworming, vaccinations, hygiene practices, and population control are essential to mitigate these risks [14]. Educating pet owners is a vital function of veterinarians in informing them about proper care, deworming schedules, and preventive strategies, including fecal examinations, tailored deworming, and hygienic practices [20]. These interventions are vital to safeguarding both pet and owner health. However, in Bangladesh, control primarily depends on anthelmintic use, underscoring the need for owner education on cost‐effective prevention strategies [21]. Limited information exists on the risk factors for parasitic infections in Bangladeshi companion animals. Further research is essential to evaluate infection risks, improve owner awareness, and optimize deworming schedules. Identifying risk factors related to pets’ living conditions, management, and diet is critical for controlling parasitism and preventing zoonotic diseases [22]. This study is aimed at collecting self‐reported data from pet owners, classifying pets into infection risk groups following ESCCAP guidelines 01, 3rd edition, July 2017, and identifying risk factors to inform effective control strategies for parasitic infections and zoonoses.

## 2. Methodology

### 2.1. Area and Population of the Study

**Figure 1:**
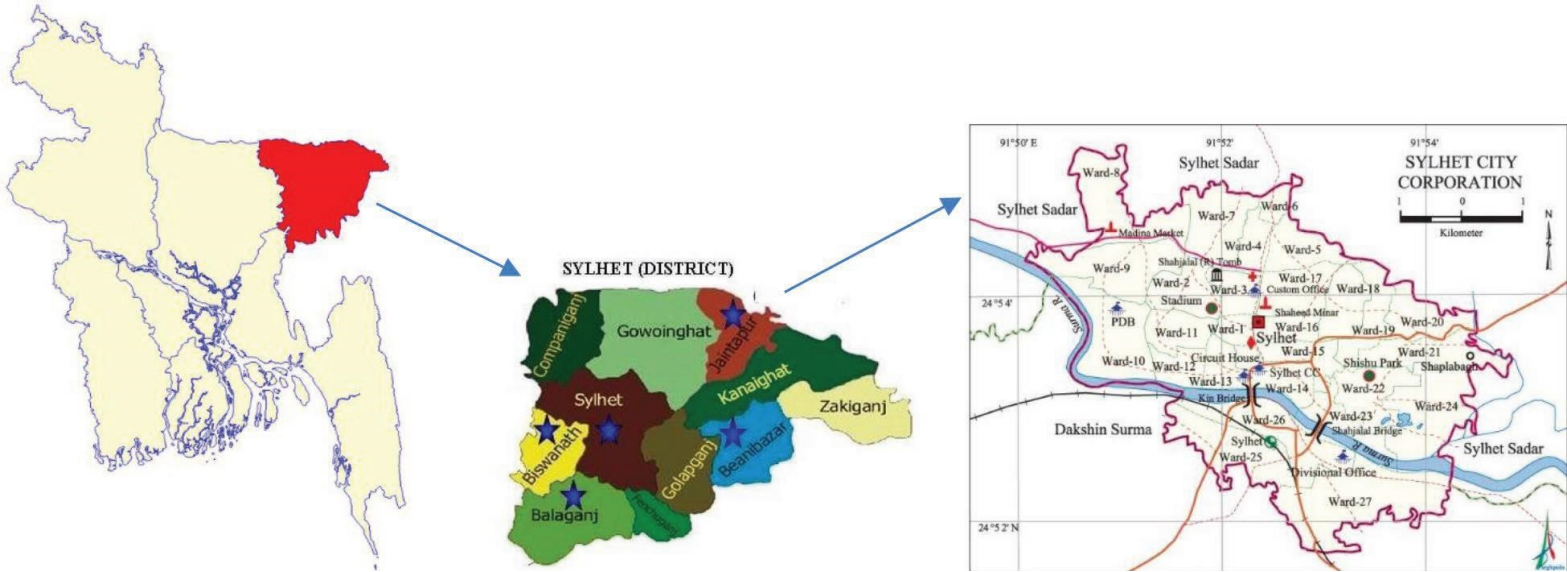
Maps of study areas.

This study, conducted in Sylhet City Corporation, Bangladesh, from September 2023 to August 2024, surveyed 229 dog and cat owners visiting Sylhet Pet Care, Vet Care Centre Sylhet, PMAC Veterinary Teaching Hospital Sylhet, Pets Cure & Care Sylhet, District Veterinary Hospital Sylhet, and Sylhet Sadar Upazila Veterinary Hospital, Bangladesh (Figure 1).

### 2.2. Design of the Study

The survey design used was cross‐sectional, and a structured offline questionnaire was used to gather data. Respondents were directly interviewed to ensure the authenticity and reliability of the information, avoiding indirect or online methods. The questionnaire was nonanonymous, recording respondent details to enable detailed data analysis. When both dogs and cats cohabited in a household, the owner was randomly categorized as either a dog or cat owner to maintain a balanced dataset to avoid double‐counting respondents in separate dog and cat datasets, although this approach could overlook unique management patterns specific to mixed‐species households.

### 2.3. Inclusion and Exclusion Criteria

Respondents must meet the following requirements to be eligible: (a) possess at least one dog or cat; (b) be at least 16 years old; (c) not be physically or mentally disabled; (d) be solely in charge of the pet’s upkeep, feeding, and veterinary visits; and (e) take their pet to the vet at least once. To ensure the respondent treats his or her dog or cat as a pet when filling out the questionnaire, the following respondents were excluded: (a) owners who had more than 10 cats; (b) professional pet owners; and (c) foster house owners, breeders, or pet animal traders [22].

### 2.4. Demographic and Household Representation

Quotas were established to ensure a representative sample of Bangladeshi pet owners based on demographic and household characteristics. These included the following: age and gender of the respondent; place of residence (rural, semiurban, or urban); household composition, such as the presence of children or pregnant women; and educational level and employment status of the respondent.

### 2.5. Risk Group

Risk group definitions according to German ESCCAP guidelines 01, 3rd edition, July 2017, for companion animals, without consideration of special risk factors (e.g., puppies, kittens, and animals used for exhibitions) [22] (Table 1).

**Table 1:** Risk group definition according to ESCCAP guidelines.

Risk group	Description	ESCCAP‐recommended deworming frequency
A	Lives indoors only or goes outdoors but has no direct contact with dogs and cats of other households and does not eat prey animals/raw meat, carrion, or feces	1–2 times per year
B	Goes outdoors under supervision and has direct contact with dogs and cats of other households but does not eat prey animals/raw meat, carrion, or feces	4 times per year
C	Goes outdoors under supervision and has direct contact with dogs and cats of other households and eats prey animals/raw meat but does not eat carrion or feces	4 times per year against roundworms, 12 times per year against tapeworms
D	Goes outdoors without supervision or under supervision but has direct contact with dogs and cats of other households and eats carrion or feces	12 times per year

We acknowledge that local parasite ecology in a South Asian region like Sylhet, Bangladesh, may differ considerably from a European environment, even though the current study uses the ESCCAP criteria as an internationally accepted risk‐based strategy in the absence of standardized local guidelines. Both endoparasite and ectoparasite transmission may be affected by elements like humidity, tropical weather, and local intermediate hosts. The results should be assessed in light of Bangladesh’s unique environmental features, even if ESCCAP provides an acceptable approach for risk rating (A–D).

### 2.6. Statistical Analysis

Based on the interview data, associations between different risk groups and potential risk factors (such as owner sociodemographic characteristics, including age, gender, education, residency, responsibility, attitude, and frequency of veterinary visits) were examined using chi‐square tests with 3 × 4 and 2 × 4 contingency tables. Statistical analyses and data visualization were conducted in GraphPad Prism Version 10.8. A significance level of *p* < 0.05 at a 95% confidence interval was considered statistically significant.

## 3. Results and Discussion

### 3.1. Pet Owners’ Sociodemographic Status

Sociodemographic status or factors of pet owners were recorded in this study. Male owners were more common than female owners in the case of dog rearing, but the opposite scenario occurred in the case of cat rearing. In terms of age, owners aged 18–24 years represented the highest proportion (50%–65%) for both dog and cat rearing, but owners aged ≥ 35 years were the least represented in dog rearing, and owners aged < 18 years were the least represented in cat rearing. Most of the pet owners were in urban areas (60%–70%), and the lowest proportion was in rural areas (6%–19%) for both dogs and cats. Attitude toward pet animals was highest for devotion (50% for dogs and 45.2% for cats) and lowest for disappointment (18% for dogs and 14% for cats). Responsibility toward pets was highest when owners were solely responsible (65% for dogs and 63.2% for cats) and lowest when pets were adopted (9.3% for dogs and 18.4% for cats). Educational qualification of the owners was highest at the graduate level among cat owners and lowest among dog owners (39.6% and 12.4%, respectively). The majority of pet owners visited a veterinarian more than once per year (56.3% of dog owners and 70% of cat owners). A household’s typical average headcount was at a minimum level (3–3.5 per family), and the average number of children (0.41–0.42 per family) was also at a minimum level compared with households of nonpet owners (details in Table 2). Previously, a few research studies conducted in Dhaka, Chattogram, and Rajshahi in Bangladesh examined the sociodemographic status of pet owners. These studies reported that 63.2% of female owners kept cats and 58% of male owners kept dogs [23]. Another study also showed that 68% of pet owners were female and only 32% were male [24]. Most of the pet owners were aged 21–30 years, whereas the fewest owners were aged < 10 years [23]. In addition, 62% of pet owners were from urban areas and 16% were from rural areas. Regarding education, 44.7%, 28%, and 18% of owners had higher secondary, secondary, and primary education, respectively [25]. Another study showed that 75% of pet owners had a bachelor’s degree or higher, and only 25% of pet owners had less than a bachelor’s degree [26].

**Table 2:** Results of the dog and cat questionnaires: Variables related to pet owners.

Variable	Dog dataset (*n* = 32)	Cat dataset (*n* = 197)
Gender, *n* (%)	Male: 20 (62%)	Male: 93 (47.2%)
	Female: 12 (38%)	Female: 104 (52.8%)
Age range (year), *n* (%)	18–24 years: 21 (65%)	18–24 years: 101 (51%)
	25–35 years: 9 (28%)	25–35 years: 43 (22%)
	35 years+: 2 (7%)	35 years+: 31 (16%)
	Under 18 years: 0%	Under 18 years: 22 (11%)
Residency, *n* (%)	Urban: 19 (60%)	Urban: 138 (70%)
	Semiurban: 7 (21%)	Semiurban: 48 (24%)
	Rural: 6 (19%)	Rural: 11 (6%)
Attitude toward a pet, *n* (%)	Devoted: 16 (50%)	Devoted: 89 (45.2%)
	Affectionate: 10 (32%)	Affectionate: 59 (30%)
	Disappointed: 6 (18%)	Disappointed: 49 (24.8%)
Number of people in the household	3.56 ± 1.32, mean ± SD	3.49 ± 1.29
Number of children in the household	0.42 ± 0.62, mean ± SD	0.41 ± 0.60
Responsibility toward a pet, *n* (%)	Sole: 21 (65%)	Sole: 125 (63.2%)
	Adopted: 3 (9.3%)	Adopted: 36 (18.4%)
	Rescued: 8 (25.7%)	Rescued: 36 (18.4%)
Visiting a veterinarian per year, *n* (%)	Single: 14 (43.7%)	Single: 60 (30%)
	Multiple: 18 (56.3%)	Multiple: 137 (70%)
Educational qualification, *n* (%)	Graduation: 4 (12.4%)	Graduation: 78 (39.6%)
	HSC: 14 (43.8%)	HSC: 71 (36%)
	Under HSC: 14 (43.8%)	Under HSC: 48 (24.4%)

Abbreviations: HSC, higher secondary school certificate; SD, standard deviation.

### 3.2. Categorized Dogs and Cats According to ESCCAP Guidelines 01, 3rd Edition, July 2017

**Figure 2:**
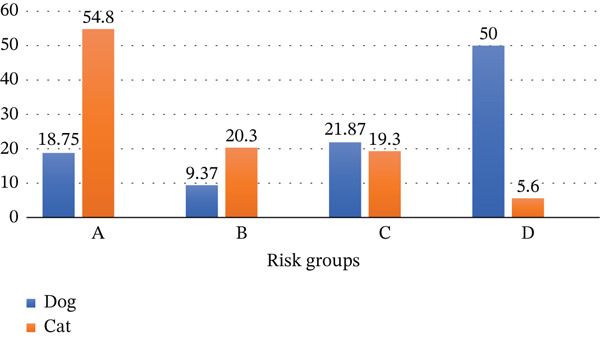
Categorization of pet animals into different risk groups according to ESCCAP guidelines 01, 3rd edition, July 2017 [22].

The highest‐risk endoparasite infection group D, for which the ESCCAP prescribes monthly deworming treatments, comprised 50% of the dog population examined in a smaller subset (*n* = 32) compared to cats (*n* = 197) in accordance with ESCCAP criteria. The majority of indoor cats (54%) were categorized as the minimum‐risk category A. The maximum danger rating D (6%) was given to the remaining outdoor cats (Figure 2). Like their German counterparts, a great deal of these risk group D cats resided in rural settings rather than cities or villages [27]. Despite being kept indoors more often than cats, a greater number of dogs were in the higher‐risk category. While just 21.8% of dogs were limited to their gardens and had no exposure to public areas like parks, sandpits, or playgrounds, a significant percentage of cats—49.74%—were documented to spend almost all of their time indoors.

A similar study conducted in Spain found the highest‐risk group D (96.21%) in the case of dogs and the highest‐risk group A (62%) in the case of cats [22]. In Bangladesh, the percentage of cats in risk group A is lower than that in Spain because cats are more often kept indoors in developed countries like Spain; normally, in Bangladesh, most of the households are not cat‐proof. Dogs in risk group D are usually more numerous than cats because of their feeding behavior and other parameters recorded in several studies [28].

### 3.3. Owners’ Sociodemographic Status Associated With Different Risk Groups

The study highlights a “theoretical risk” and suggests that future research incorporating parasitological testing (e.g., fecal examinations) is needed to correlate these risk levels with actual infection prevalence. Our study showed an association between owners’ sociodemographic status and different risk groups. Variables such as area of residency, attitude, educational qualification, visits to the vet per year, pet rearing, responsibility toward a pet, and number of pets in the household are mentioned separately in Tables 3 and 4. Most of the pet owners were from urban areas, and the fewest owners were from rural areas, with some from semiurban areas. In the case of dogs from risk group A, most of them belonged to city dwellers (60%), and the fewest were from rural areas (19%) (Table 3). Similarly, for cats from risk group A, most of them belonged to city dwellers, and the fewest were also from rural areas (Table 4). Pet keeping is often popular in urban areas compared to rural areas due to several factors, such as lifestyle, social isolation, housing constraints, convenience, and culture [29]. The association between area of residency (urban, semiurban, and rural) of pet owners and ESCCAP infection risk groups (A, B, C, and D) was moderately significant for dogs (*p* < 0.05) (Table 3) but significant for cats (*p* < 0.05) (Table 4). In the case of attitude, most owners are devoted to their pets because pets provide emotional support, reduce stress, and improve well‐being; taking care of a pet gives owners a sense of purpose and responsibility and ultimately helps owners keep social connections [30–33]. Owners’ attitudes (devoted, affectionate, and disappointed) toward pets were not significantly associated with different risk groups in dogs (*p* > 0.05) (Table 3) but were highly significant in the case of cats (*p* < 0.005) (Table 4) though most of the owners were classified as devoted and affectionate, and the fewest were classified as disappointed. Attitudes toward pets can influence the level of care they receive, which in turn may impact the risk of parasitic infection in companion animals. Positive attitudes toward pet care, including regular veterinary visits, parasite prevention, and proper hygiene practices, have been connected to preventive healthcare practices, an important aspect of the One Health concept for minimizing the spread of zoonotic diseases. A study investigated the relationship between pet owners’ attitudes toward preventive healthcare and the prevalence of parasitic infections in dogs. The study found that dogs owned by individuals with positive attitudes toward preventive healthcare, including regular deworming and flea control, had lower rates of parasitic infections compared to dogs owned by individuals with less proactive attitudes [34]. This suggests that pet owners’ attitudes and behaviors toward pet care can play a significant role in reducing the risk of parasitic infections in companion animals. Educational qualification of the dog owners here showed that the highest percentage had passed higher secondary education; in the case of risk group D, no owners were graduates (0%), and the highest percentage had education under HSC (100%; 6/6) (Table 3). Educational qualification of the dog owners was moderately significantly associated with the different risk groups (*p* < 0.05); in the case of cat owners, education was not significantly associated with the different risk groups (*p* > 0.05) (Table 4). Owners of pets generally visit veterinary hospitals or veterinarians multiple times a year; they are devoted to their pets, and some owners only see the vet for health checkups. Visits to vets were moderately significantly associated with the risk groups. The maximum‐risk group (D) showed the highest frequency of vet visits, and the minimum‐risk group (A) showed the lowest frequency for both dogs and cats (*p* < 0.05) (Tables 3 and 4).

**Table 3:** Dog owners’ sociodemographic factors associated with different risk groups.

Demography	Specificity	Risk group A *n*(%)	Risk group B *n*(%)	Risk group C *n*(%)	Risk group D *n*(%)	Total *N*(%)	Significance
Residency	Urban	5 (26.3%)	2 (10.5%)	1 (5.3%)	11 (57.9%)	19 (100%)	*χ* ^2^ = 11.82, df = 6, *p* < 0.05
	Semiurban	0 (0.0%)	1 (14.3%)	3 (42.8%)	3 (42.9%)	7 (100%)	
	Rural	1 (16.7%)	0 (0.0%)	3 (50.0%)	2 (33.3%)	6 (100%)	
	Total	6 (18.8%)	3 (9.4%)	7 (21.9%)	16 (50.0%)	32 (100%)	
Attitude	Devoted	1 (6.25%)	3 (18.75%)	3 (18.75%)	9 (56.25%)	16 (100%)	*χ* ^2^ = 4.54, df = 6, *p* > 0.05
	Affectionate	2 (20%)	0 (0.0%)	2 (20%)	6 (60%)	10 (100%)	
	Disappointed	1 (16.67%)	0 (0.0%)	2 (33.33%)	3 (50%)	6 (100%)	
Education	Graduation	0 (0.0%)	1 (25.0%)	3 (75.0%)	0 (0.0%)	4 (100%)	*χ* ^2^ = 15.31, df = 6, *p* < 0.05
	HSC	2 (14.3%)	8 (57.1%)	4 (28.6%)	0 (0.0%)	14 (100%)	
	Under HSC	3 (21.4%)	3 (21.4%)	2 (14.3%)	6 (42.9%)	14 (100%)	
Gender	Male	1 (5.0%)	3 (15.0%)	6 (30.0%)	10 (50.0%)	20 (100%)	*χ* ^2^ = 8.78, df = 3, *p* < 0.05
	Female	5 (41.7%)	0 (0.0%)	1 (8.3%)	6 (50.0%)	12 (100%)	
Vet visit/year	Single	1 (7.1%)	2 (14.3%)	4 (28.6%)	7 (50.0%)	14 (100%)	*χ* ^2^ = 7.94, df = 3, *p* < 0.05
	Multiple	5 (27.8%)	1 (5.6%)	0 (0.0%)	12 (66.7%)	18 (100%)	
Responsibility	Sole	4 (19.05%)	2 (9.52%)	4 (19.05%)	11 (52.38%)	21 (100%)	*χ* ^2^ = 5.16, df = 6, *p* > 0.05
	Adopted	1 (33.33%)	1 (33.33%)	1 (33.33%)	0 (0.0%)	3 (100%)	
	Rescued	1 (12.5%)	0 (0.0%)	2 (25%)	5 (62.5%)	8 (100%)	
Age	18–24 years	5 (23.81%)	3 (14.29%)	4 (19.05%)	9 (42.86%)	21 (100%)	*χ* ^2^ = 4.83, df = 6, *p* > 0.05
	25–35 years	1 (11.11%)	0 (0.0%)	3 (33.33%)	5 (55.56%)	9 (100%)	
	35 years+	0 (0.0%)	0 (0.0%)	0 (0.0%)	2 (100%)	2 (100%)	

Abbreviation: HSC, higher secondary school certificate.

**Table 4:** Cat owners’ sociodemographic factors associated with different risk groups.

Demography	Specificity	Risk group A	Risk group B	Risk group C	Risk group D	Total *N*(%)	Significance
Residency	Urban	82 (59.4%)	26 (18.8%)	23 (16.7%)	7 (5.1%)	138 (100%)	*χ* ^2^ = 17.66, df = 6, *p* < 0.05
	Semiurban	23 (47.9%)	14 (29.2%)	9 (18.8%)	2 (4.2%)	48 (100%)	
	Rural	3 (27.3%)	0 (0.0%)	6 (54.5%)	2 (18.2%)	11 (100%)	
	Total	108 (54.8%)	40 (20.3%)	38 (19.3%)	11 (5.6%)	197 (100%)	
Attitude	Devoted	57 (64.0%)	24 (27.0%)	6 (6.7%)	2 (2.3%)	89 (100%)	*χ* ^2^ = 105.6, df = 6, *p* < 0.005
	Affectionate	2 (3.4%)	38 (64.4%)	11 (18.6%)	8 (13.6%)	59 (100%)	
	Disappointed	13 (26.5%)	3 (6.1%)	14 (28.6%)	19 (38.8%)	49 (100%)	
Education	Graduation	44 (56.41%)	17 (21.79%)	14 (17.99%)	3 (3.85%)	78 (100%)	*χ* ^2^ = 2.42, df = 6, *p* > 0.05
	HSC	38 (53.52%)	16 (22.54%)	12 (16.90%)	5 (7.04%)	71 (100%)	
	Under HSC	27 (56.25%)	7 (14.58%)	11 (22.92%)	3 (6.25%)	48 (100%)	
Gender	Male	50 (55.56%)	19 (21.11%)	19 (21.11%)	5 (5.56%)	90 (100%)	*χ* ^2^ = 0.17, df = 3, *p* > 0.05
	Female	58 (55.77%)	21 (20.19%)	19 (18.27%)	6 (5.77%)	104 (100%)	
Vet visit/year	Single	27 (45.0%)	10 (16.7%)	17 (28.3%)	6 (10.0%)	60 (100%)	*χ* ^2^ = 8.75, df = 3, *p* < 0.05
	Multiple	81 (59.1%)	30 (21.9%)	21 (15.3%)	5 (3.7%)	137 (100%)	
Responsibility	Sole	78 (62.4%)	30 (24.0%)	14 (11.2%)	3 (2.4%)	125 (100%)	*χ* ^2^ = 12.59, df = 6, *p* < 0.05
	Adopted	24 (66.7%)	7 (19.4%)	4 (11.1%)	1 (2.8%)	36 (100%)	
	Rescued	16 (44.4%)	6 (16.7%)	11 (30.6%)	3 (8.3%)	36 (100%)	
Age	Under 18 years	13 (59.09%)	4 (18.18%)	4 (18.18%)	1 (4.55%)	22 (100%)	*χ* ^2^ = 7.74, df = 9, *p* > 0.05
	18–24 years	57 (56.44%)	20 (19.80%)	21 (20.79%)	3 (2.97%)	101 (100%)	
	25–35 years	22 (51.16%)	12 (27.91%)	6 (13.95%)	3 (6.98%)	43 (100%)	
	35 years+	16 (51.61%)	4 (12.90%)	7 (22.58%)	4 (12.90%)	31 (100%)	

Abbreviation: HSC, higher secondary school certificate.

Responsibility toward pets was highest when owners had sole responsibility and lowest when pets were adopted for both dogs and cats. The minimum‐risk group (A) was usually associated with sole responsibility, and the maximum‐risk group (D) was associated with disappointed responsibility, as recorded in this study. Responsibility of pet owners was highly significantly associated with the ESCCAP risk groups (*p* < 0.05) (Tables 3 and 4). Sole responsibility or sole ownership of a cat can potentially reduce the risk of parasitic infection compared to multiple ownership situations, where cats may have increased opportunities to be exposed to parasites. Recently, several research studies have shown that the educational qualification of pet owners is crucial for managing parasitic infections in animals and that owners’ perceptions of zoonotic risks differ based on factors like gender, pet ownership duration, and frequency of veterinary visits [35]. Despite veterinary recommendations for regular anthelmintic treatment, many pets, especially those at higher risk, are dewormed less often than suggested [23]. This may stem from insufficient owner education about zoonotic risks, as veterinarians often neglect to discuss the topic [36]. Increased risk factors for parasitic infections include feeding raw viscera, scavenging, inadequate deworming, and poor owner education; other risk factors include the pet’s age, gender, living conditions, and environment [37, 38]. Preventing zoonotic infections requires a One Health approach integrating efforts from veterinary and medical professionals, alongside public health personnel. Improved communication between these groups could enhance prevention and treatment strategies [16, 39].

### 3.4. Variables Related to the Common Understanding of Pet Owners

**Figure 3:**
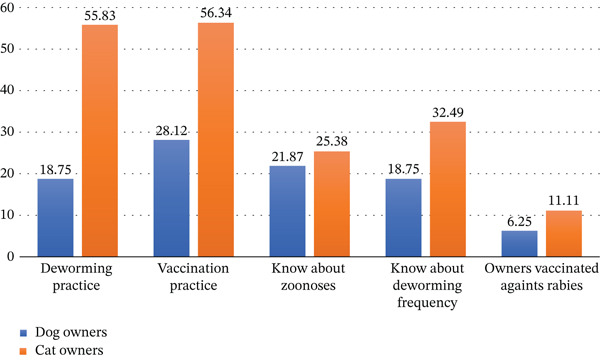
Pet owners’ understanding of different matters related to keeping pet animals.

Variables related to the common understanding of pet owners included knowledge of annual deworming frequency, regular vaccination, awareness of zoonotic diseases, rabies vaccination of owners, etc. Among cat owners, 56.3% (111/197) practiced regular vaccination of their cats, whereas only 28.12% (9/32) of dog owners practiced regular vaccination of their dogs. These results clearly show that cat owners were more aware than dog owners about vaccination (Figure 3). In a previous study conducted in Dhaka and Chattogram, 62.5% of dog owners practiced vaccination for their beloved dogs [23]; in another study conducted in Dhaka, Bangladesh, 66.13% of cat owners practiced vaccination regularly [40]. Only 21.87% of dog owners and 25.38% of cat owners had heard about zoonotic diseases; most of the pet owners had no idea about zoonoses. In 2019, a previous study was conducted in the coastal area of Barguna District, Bangladesh, on knowledge, attitude, and practice toward zoonotic diseases among different professionals. That study found that 37.5% of people knew about zoonoses [41]. Only 6.25% of dog owners and 11.11% of cat owners were recorded as vaccinated against rabies in this study. It was also recorded that only 18.75% of dog owners knew the normal deworming frequency, whereas 32.49% of cat owners knew the normal deworming frequency. Despite having high veterinary visit rates, deworming compliance among dog owners remained suboptimal (18.75%), highlighting a failure in owner education regarding specific regimens and adherence. But, in the case of cat owners, 55.83% (minimum of once per year) have been recorded in this study. As previously mentioned, a study in Dhaka on the health status of cats found that 59.68% of cat owners dewormed their pets (minimum of once per year) [40]. In contrast to our study, 50% of dog owners dewormed their pets (minimum of once per year), as recorded in a recent survey on pet animals in Dhaka and Chattogram [23].

## 4. Conclusion

This study examines the link between pet owners’ sociodemographic factors and infection risks in cats and dogs in Sylhet City, Bangladesh. Cats were mostly at low risk due to indoor living, while dogs faced higher risks from outdoor exposure. Factors such as age, education, and attitudes influenced preventive care. However, awareness of zoonotic diseases was low, especially among dog owners. The findings highlight the need for better health education, regular veterinary care, and a One Health approach to reduce zoonotic risks and improve both animal and human health.

## 5. Limitations

In Bangladesh, including Sylhet City, the majority of companion animals are cats rather than dogs. Consequently, this study included 197 cats and only 32 dogs. The statistical analysis becomes restricted by this unequal sample size, which additionally reflects the real pet ownership characteristics in Sylhet, where cat ownership is considerably more common. Recall and social desirability bias should be taken into account when interpreting the results since the data were self‐reported and gathered through nonanonymous interviews to support comprehensive research. As there are currently no such standardized parasite control guidelines specifically for Bangladesh, we applied ESCCAP GL1 in a South Asian setting. We assessed pets in relation to behaviors and husbandry practices known to influence parasite exposure using the ESCCAP framework, an internationally approved risk‐based starting point, in the absence of local rules and regulations. We understand that these risk classifications are not indicators of verified infection status; they are based on owner‐reported behavior rather than diagnostic testing and reflect potential exposure. Although our study focused on owner‐reported risk factors and sociodemographic influences, we highlighted that this study serves as a foundational assessment to inform much‐needed local education and the eventual development of regional standards of practice. We agree that diagnostic validation (e.g., fecal analysis) is a crucial next phase that was absent here. Yet, even in cases where clinical infection status is unknown, we used the ESCCAP risk assessment framework, a recognized clinical technique for placing dogs into risk groups (A–D), to estimate optimal deworming frequencies.

## Author Contributions

Saiful Islam acted as the research supervisor. Saiful Islam, Rakibul Hasan, Shofiqul Islam, Md. Khairul Amin Rafi, Md. Saruar Jahan Nayem, Onnapurna Roy, Tushar Kanti, Marjana Mowrin Jui, Muhammad Mujahidul Islam, Mohammad Sujaur Rahman, Md. Mowdudul Hasan Talha, Real Datta, Kazi Mehetazul Islam, and Saiful Islam carried out the study design, field experiments, data analysis, and manuscript writing. Saiful Islam, Kazi Mehetazul Islam, and Saiful Islam assisted with morphometrical analysis and writing. Saiful Islam revised the paper. Saiful Islam and Saiful Islam both made equal contributions to this piece of work.

## Funding

No funding was received for this manuscript.

## Conflicts of Interest

The authors declare no conflicts of interest.

## Data Availability

Upon appropriate request, the corresponding author and the Department of Parasitology, Sylhet Agricultural University, Bangladesh, will make the documents used for the present research available.
